# Genomic and Biocontrol Potential of the Crude Lipopeptide by *Streptomyces bikiniensis* HD-087 Against *Magnaporthe oryzae*

**DOI:** 10.3389/fmicb.2022.888645

**Published:** 2022-06-09

**Authors:** Wei Liu, Jiawen Wang, Shan Li, Huaqian Zhang, Li Meng, Liping Liu, Wenxiang Ping, Chunmei Du

**Affiliations:** ^1^Engineering Research Center of Agricultural Microbiology Technology, Ministry of Education, Heilongjiang University, Harbin, China; ^2^Key Laboratory of Microbiology, College of Heilongjiang Province, School of Life Sciences, Heilongjiang University, Harbin, China

**Keywords:** biological control, lipopeptide, *Magnaporthe oryzae*, *Streptomyces bikiniensis*, whole genome

## Abstract

Rice blast caused by *Magnaporthe oryzae* is one of the most destructive plant diseases. The secondary metabolites of *Streptomyces* have potential as biological control agents against *M. oryzae*. However, no commercial secondary antimicrobial products of *Streptomyces* have been found by gene prediction, and, particularly relevant for this study, a biocontrol agent obtained from *Streptomyces bikiniensis* has yet to be found. In this research, genomic analysis was used to predict the secondary metabolites of *Streptomyces*, and the ability to develop biocontrol pharmaceuticals rapidly was demonstrated. The complete genome of the *S. bikiniensis* HD-087 strain was sequenced and revealed a number of key functional gene clusters that contribute to the biosynthesis of active secondary metabolites. The crude extract of lipopeptides (CEL) predicted by NRPS gene clusters was extracted from the fermentation liquid of *S. bikiniensis* HD-087 by acid precipitation followed by methanol extraction, and surfactins, iturins, and fengycins were identified by liquid chromatography-mass spectrometry (LC–MS). *In vitro*, the CEL of this strain inhibited spore germination and appressorial formation of *M. oryzae* by destroying membrane integrity and through the leakage of cellular components. *In vivo*, this CEL reduced the disease index of rice blast by approximately 76.9% on detached leaves, whereas its control effect on leaf blast during pot experiments was approximately 60%. Thus, the *S. bikiniensis* CEL appears to be a highly suitable alternative to synthetic chemical fungicides for controlling *M. oryzae*.

## Introduction

Rice (*Oryza sativa*) is an important food crop and the staple diet of over three billion people around the world (Woan-Fei et al., 2017). However, rice blast disease caused by *Magnaporthe oryzae* is a serious threat to rice production and can cause yield losses of 10%–35% per year (Li et al., 2021). Currently, the main agents used to control rice blast are still chemical fungicides (Zhou et al., 2021). However, the excessive use of such fungicides over the years is not only responsible for the development of resistance in the target fungal strains but also causes disruption of the microbial community, leading to decreased soil health (Meyer et al., 2021) and not favoring the meeting of sustainable agricultural development goals. Thus, the use of biological control agents to replace chemical pesticides has been an important research topic in the field of rice production because these agents possess various suitable properties, such as security, easy degradation, and good environmental compatibility.

Currently, both living organism and metabolite preparations of bacteria, fungi, and actinomycetes against rice blast have been studied, but only a few agents have been applied to rice production (Chakraborty et al., 2021). Among them, the control effects of living organism agents are typically influenced by many unknown factors, such as changeable environmental and ecological factors, and cannot produce ideal results (Ali et al., 2016). Microbial metabolite preparations have some relatively extraordinary advantages, such as a rapid response, stable effect and long shelf life, and have become, therefore, a specific area of focus of biological control in the field. In particular, the metabolites of *Streptomyces* are so rich and multifarious that they have been regarded as a high-quality resource library for the development of biocontrol agents and can be mined for a variety of compounds to protect plants from pathogens, such as antimicrobial compounds and plant growth-promoting substances (Qi et al., 2019). For example, blasticidin-S produced by the soil actinomycete *Streptomyces griseochromogenes* (He et al., 2019) and kasugamycin produced by *Streptomyces kasugaensis* have played an important role as classical commercial biocontrol agents against rice blast (Napolioni et al., 2019).

However, due to more than 70 years of repeated isolation and screening of most actinomycetes and the continual isolation of bioactive natural products, the probability of finding new active compounds has become increasingly difficult (Li et al., 2018). Microorganism genome sequencing provides an opportunity to identify important antimicrobial compounds, discover more new secondary metabolites and extract non-toxic antimicrobial substances in a more targeted manner (Jing et al., 2020). Thus, analyses to predict the gene functions of the actinomycetes genome may be able to maximize the production potential of secondary metabolism through the genetic manipulation of synthetic or regulatory genes. Until now, none of the commercial antimicrobial products have been found by gene prediction; blind screening has typically been used, but this approach generally requires substantial time and human-resource investment.

*Streptomyces bikiniensis* HD-087 was isolated from agricultural soil in Hulunbuir, in the Inner Mongolia Autonomous Region, China (Zhao et al., 2012). Its fermentation solution has been found to disrupt the cell membrane and significantly inhibit mycelium growth of *Fusarium oxysporum* HU-M, the pathogen responsible for cucumber wilt (Zhao et al., 2014). However, the effective antifungal components in the metabolites of *S. bikiniensis* HD-087 have yet to be clarified, and no industrial agent derived from *S. bikiniensis* for use in agriculture has been developed so far. To exploit the biocontrol potential of *S. bikiniensis* HD-087, its whole genome was sequenced, and some gene clusters for synthesizing antibiotic metabolites were annotated. Then, based on the gene prediction results, various secondary metabolite substances were extracted and identified by LC–MS. Subsequently, the effectiveness of the extractive matter against *M. oryzae* was revealed by controlled experiments *in vivo* and *in vitro*, laying the foundation for their application in rice blast control. This study highlights that using genomic analysis to predict and develop biocontrol pharmaceutical is a feasible strategy.

## Materials and Methods

### Test Strain, Culture Medium, and Rice Variety

*Streptomyces bikiniensis* HD-087 was isolated, screened, identified, and preserved by the Microbiology Laboratory of Heilongjiang University. *Magnaporthe oryzae* Guy11 was donated by Dr. Chong Zhang of Shenyang Agricultural University, Liaoning province, China. It was grown on Gauze’s synthetic broth medium no. 1 (Shi et al., 2021) for activation and then inoculated into DBY medium (glucose 20 g·L^−1^, soybean flour 5 g·L^−1^, yeast flour 4 g·L^−1^, ammonium sulfate 5 g·L^−1^, NaCl 1 g·L^−1^, and K_2_HPO_4_ 0.05 g·L^−1^) for lipopeptide production. *Magnaporthe oryzae* Guy11 was routinely cultured on PDA (B; Virna et al., 2010) medium, and oatmeal-tomato juice agar medium (OMTA; Nie et al., 2014) was used for spore production. The rice variety was Long Japonica 46, one of the main cultivars in Heilongjiang Province, China.

### Genome Sequencing and Metabolite Prediction

The draft genome sequence of *S. bikiniensis* HD-087 investigated in the present study was submitted to the National Center for Biotechnology Information NCBI GenBank under accession number PRJNA823498. The whole-genome sequencing and assembly of *S. bikiniensis* HD-087 was performed by Megabio. Glimmer (version 3.02) software was used for gene predictions of the assembly results and visualized by the CGView Server. The rRNA was predicted with Barrnap (version 0.8) software, and the tRNA region and the secondary structure of tRNA were predicted with tRNA-scan-SE (version 2.0) software. GO (Gene Ontology) was annotated with blast2go, Diamond comparison was used to perform the Clusters of Orthologous Groups of proteins (COG) annotation, and the Kyoto Encyclopedia of Genes and Genomes (KEGG) and annotation databases were used to complete the protein sequence function annotation. The biosynthetic gene clusters of the secondary metabolites were predicted using the online antiSMASH v4.2.0 software (Huang et al., 2019).

### Preparation of the Crude Extracts of Lipopeptide and Identification of the Active Ingredient

*Streptomyces bikiniensis* HD-087 was inoculated in Gauze’s synthetic broth medium no. 1 and cultured in a shaking incubator at 180 r·min^−1^ and 28°C for 3 days. Then, 2 ml of culture solution was inoculated into a 250 ml flask containing 50 ml of DBY medium and incubated on a shaker at 180 r·min^−1^ for 4 days at 28°C, and fermentation broth was obtained. Crude extract of lipopeptides (CEL) was extracted from the fermentation broth by acid precipitation and alcohol extraction according to the method of Gong et al. (2014) and then analyzed by LC–MS Q-TOF (LC–MS 6545, Agilent, United States) at 220 nm with a C18 column (150 mm × 4.6 mm, 5 μm). The mobile phase was a mixture of acetonitrile and ultrapure water (4:6, v/v) with a 10 μl injection volume of samples.

### Effect of CEL on Spore Germination and Appressorium Formation of *Magnaporthe oryzae*

Five copies in 50 μl of *M. oryzae* spore suspension (approximately 20–40 spores per field of vision) were added to sterilized concave slides (Liu et al., 2017), and then equal amounts of CEL solution were added at five different concentrations (i.e., 200, 100, 50, 25, and 0 μg·ml^−1^), mixed well and cultured at 28°C for 12, 24, 36 and 48 h, then sampled and observed with a microscope according to Lau and Hamer (1998). Each treatment was replicated three times, and 200 spores were examined randomly in each treatment (when the budding tube was larger than the short radius of the spore, germination was considered to have taken place). The spore germination rate, spore germination inhibition rate and appressorium formation inhibition rate were calculated as follows:


$$\begin{array}{l} \mathrm{Spore germination rate}\;\left(\%\right)\\ =\frac{\mathrm{Number of spore germination}}{\mathrm{Number of spores examined}\;\mathrm{by}\;\mathrm{microscopy}}\times 100 \end{array}$$

$$\begin{array}{l} \mathrm{Inhibition rate of spore formation}\;\left(\%\right)\\ =\frac{\left[\begin{array}{l} \left(\mathrm{Spore germination number of control group}\right)\\ -\left(\mathrm{Spore germination number of treatment group}\right) \end{array}\right]}{\mathrm{Spore germination number of control group}}\times 100 \end{array}$$

$$\begin{array}{l} \mathrm{Inhibition rate of appressorium formation}\;\left(\%\right)\\ =\frac{\left[\begin{array}{l} \left(\mathrm{Appressorium formation number of control group}\right)\\ -\left(\mathrm{Appressorium formation number of treatment group}\right) \end{array}\right]}{\mathrm{Appressorium formation number of control group}}\\ \times 100 \end{array}$$

### Effect of CEL on the Cell Membrane Integrity of Mycelium and Spores of *Magnaporthe oryzae*

The mycelium test was conducted as follows: the *M. oryzae* spore suspension was adjusted to 1 × 10^5^ spores·ml^−1^, and then 3 ml was inoculated into PDB medium and shaken at 28°C for 48 h with a rotational speed of 180 r·min^−1^. Then, CEL was added to a final concentration of the EC_50_ (median effective concentration), whereas the control was free of CEL. After the CEL acted for 6 h, a stock solution of the fluorescent dye propidium iodide (PI) was added to a final concentration of 2.5 μg·ml^−1^. The samples were stained for 30 min at room temperature while avoiding light and then centrifuged at 5,000 r·min^−1^ for 3 min, washed with phosphate buffer to suspend the mycelium, and temporary slides were made and observed under the green excitation light of a fluorescence microscope. The spore test was the same as the mycelium test but did not require culture.

### Effect of CEL on *Magnaporthe oryzae* Mycelial Morphology

Two copies of *M. oryzae* mycelium suspension were prepared as previously mentioned. CEL was added to the experimental group to a final concentration of the EC_50_, and the control group was treated with the same equivalent of sterile water. A small amount of mycelium was picked at 6, 12 and 24 h after treatment, dehydrated and fixed according to the method of Li et al. (2020) and ultimately observed using scanning electron microscopy (SEM).

### Experiments on the Control of Leaf Blast on Rice Leaves *in vitro* by CEL

Rice seeds of Long Japonica 46 were sowed in pots and incubated at 28°C with a 12 h photoperiod and 80% relative humidity in a light incubator until the seedlings grew to the four-leaf-one-shoot stage. Then, the leaves were picked and sprayed with *M. oryzae* spore suspension at a concentration of 1 × 10^5^ spores·ml^−1^ with 0.02% Tween 20 as the spreading agent. Twenty-four hours later, the inoculated leaves were sprayed evenly with CEL at concentrations of 400, 200, 100, 50 and 25 μg·ml^−1^, with equal amounts of clear water as a control. All the treated leaves were cultured in a bioclimatic chamber at 28°C with 95% humidity and in the dark for the first 24 h, followed by a 12/12 h light/dark cycle. The disease index was assessed at 5 days after spraying fungicide (Tokpah et al., 2016). The incidence of rice blast was scored using a scale of 0–5 as follows: (0) no symptoms; (1) typical blast lesions with elliptical shapes measuring 1–2 cm long and usually confined to the area of the two main veins and infecting <4% of the total leaf area; (2) typical blast lesions infecting 4%–25% of the leaf area; (3) typical blast lesions infecting 26%–50% of the leaf area; (4) typical blast lesions infecting 51%–75% of the leaf area; and (5) all leaves dead (Liu et al., 2021). This experiment was repeated three times.

The disease index and biocontrol efficacy were calculated as follows:


$$\begin{array}{l} \mathrm{Disease index}\;\left(\%\right)\\ =\frac{\left[\begin{array}{l} \sum \left(\mathrm{The number of diseased plants in each disease rating}\right)\\ \times \left(\mathrm{The number of plants}\;\mathrm{at}\;\mathrm{corresponding rating}\right) \end{array}\right]}{\left(\begin{array}{l} \mathrm{Total number of plants invertigated}\\ \times \mathrm{The highest disease rating} \end{array}\right)}\\ \times 100 \end{array}$$

$$\begin{array}{l} \mathrm{Biocontrol efficacy}\;\left(\%\right)\\ =\frac{\left[\begin{array}{l} \left(\mathrm{Relative disease index of control treatment}\right)\\ -\left(\mathrm{Disease index of treatment}\right) \end{array}\right]}{\mathrm{Disease index of control}}\times 100 \end{array}$$

### Experiments on Controlling Leaf Blast on Potted Rice by CEL

The inoculation method with *M. oryzae*, the application method of the pesticide and the culture conditions of the potted rice were the same as the previous experiments on rice leaves *in vitro*. Five treatments were established as follows: (1) clear water (incidence control); (2) 2% kasugamycin (Aino Spring Thunder) as a biological agent control; (3) 85% isoprothiolane (North American Nongda); (4) tricyclazole (Mindleader) as chemical agent controls; (5) treatment with CEL at 200 μg·ml^−1^. Each treatment consisted of three replicates comprising 15 rice plants per replicate, and the disease incidence was observed and recorded at 5 days after spraying fungicide; the disease index was calculated as mentioned above (Tokpah et al., 2016).

### Statistics and Data Analysis

All the above experiments were repeated three times, and consistent data were observed. Data from representative samples were analyzed by one-way analysis of variance, and treatment means were then calculated using the least significance difference (LSD) test at a significance level of *p* < 0.05.

## Results

### Genome Sequencing and Metabolite Prediction

**Figure 1:**
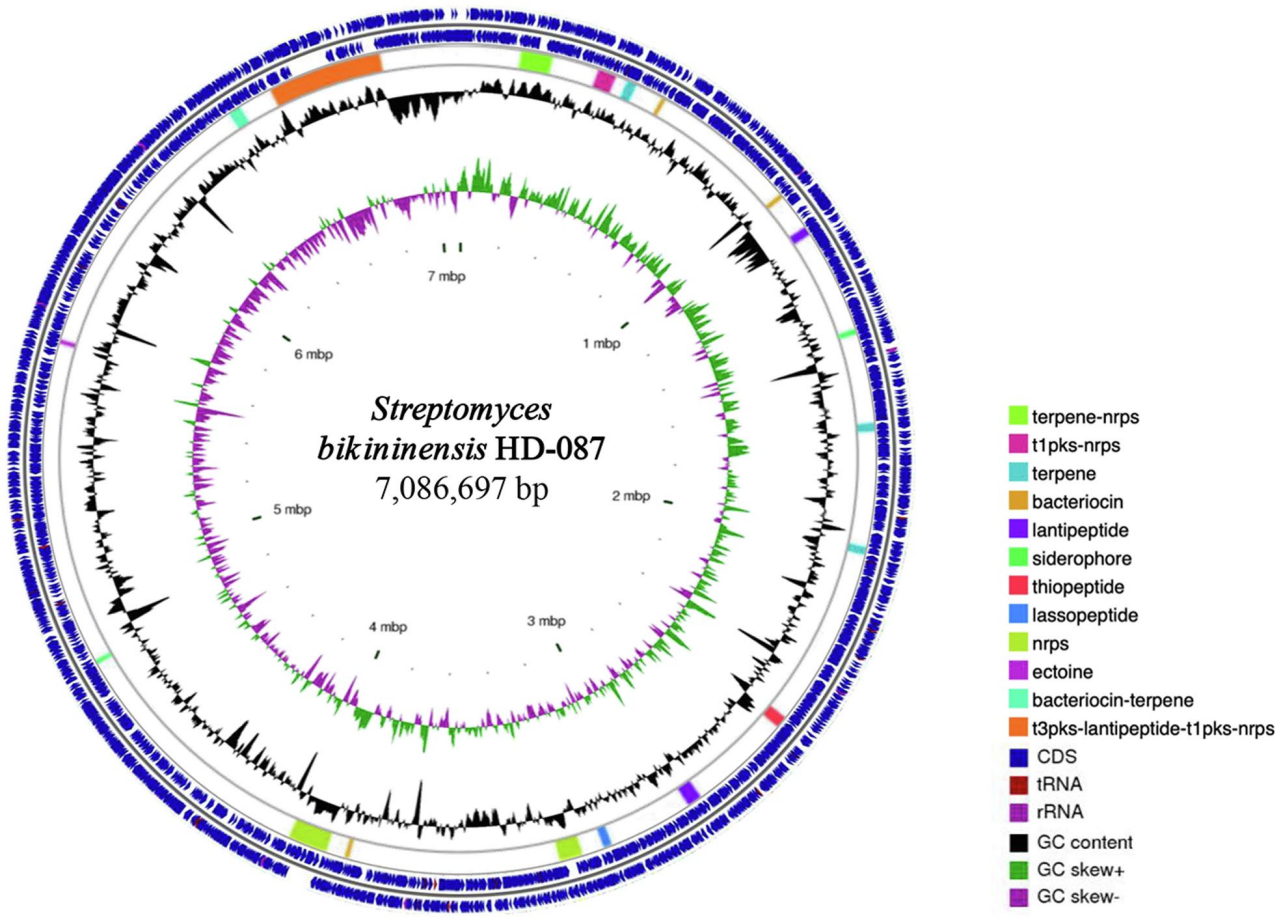
Circular genome of strain HD-087 with specific features. From the outside to the inside of the circle diagram, the first and second circles are CDS, tRNA, and rRNA on the positive and negative chains, respectively. The third circle is the distribution of gene clusters. The fourth circle is GC content. The fifth circle is the GC-SKEW value. The innermost circle indicates the size of the genome.

Following the sequencing and assembly, the genome of *S. bikiniensis* HD-087 comprised one circular chromosome of 7,086,697 bp with 73.23% GC content, including 6,536 genes, 236 tandem repeats, 64 tRNAs, 21 rRNAs, 235 minisatellite sequences, and 67 microsatellite sequences. The alignment analysis with antiSMASH software in GenBank showed that the genome of *S. bikiniensis* HD-087 contains 19 biosynthetic gene clusters encoding secondary metabolites (Figure 1), five of which encode non-ribosomal peptide synthases (NRPS), accounting for 26.23% of the total predicted gene clusters of secondary metabolite; others are most likely involved in the synthesis of terpenes, bacteriocins, lantipeptides, siderophores, thiopeptide, lassopeptides, ectoine, bacteriocin-terpenes, and some other unknown metabolites. By alignment of antiSMASH software and GenBank, more than 70% of similar gene clusters producing nine chemicals were annotated in Supplementary Table 1, including seven gene clusters with 100% similarity. In addition, 10 gene clusters cannot match well with lower similariy of 11%, these unique clusters indicate that *S. bikiniensis* HD-087 has strong potential to produce new antibiotics and is worth investigating in future work. As is well known, the NRPS gene cluster primarily controls and regulates the synthesis of lipopeptides and peptides and is also occasionally involved in the synthesis of polyketones; lipopeptides were selected as the main research object in this experiment.

### Comparative Genomics of Database

**Figure 2:**
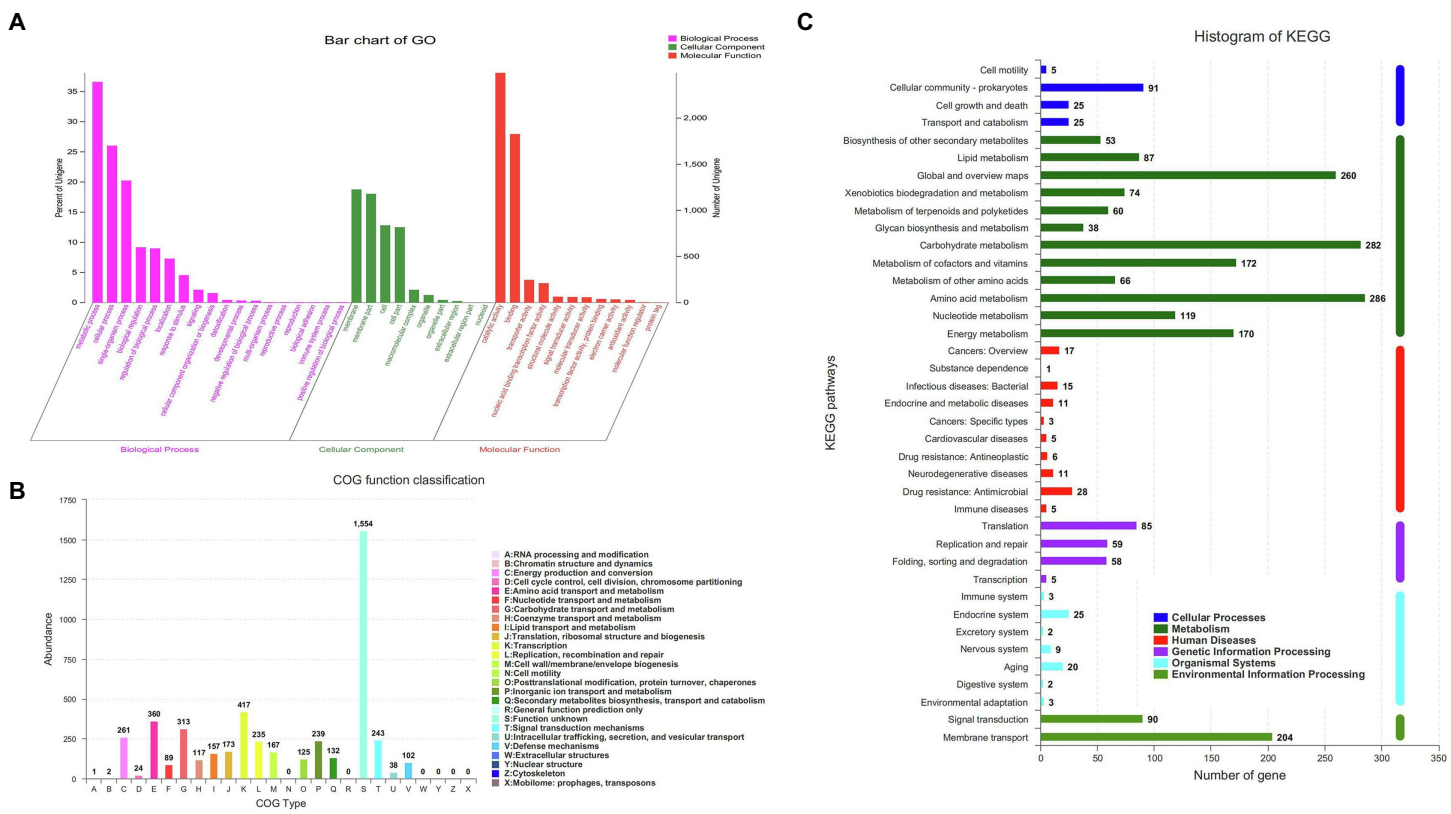
Analysis of genome structure and metabolic pathway of strain HD-087. **(A)** Go annotation of strain HD-087 genome; **(B)** COG annotation of strain HD-087 genome; **(C)** pathway annotation of strain HD-087.

A total of 4,249 genes were annotated in the GO database and could be divided into three subcategories according to the function of each gene: biological process (18 branches), cellular component (10 branches), and molecular function (12 branches; Figure 2A). In the biological process subcategories, the three largest branches are metabolic, cell and single-organism processes. In the cellular component subcategories, the branch regarding the cell membrane accounts for the largest proportion. In the molecular function subcategories, the top three are the catalytic activity, binding and transporter activity branches successively. Among a total of 9,767 identified protein-coding genes, 43.5% (4,249) and 23.3% (2,274) were annotated into COG and KEGG functional categories, respectively. For the COG categories, the highest ratio was for biological processes (45%), followed by molecular function (30%) and cellular components (25%; Figure 2B). The abundance of unknown function was found to be the highest, followed by transcription, amino acid transport and metabolism and carbohydrate transport and metabolism, with the abundance of other subcategories being below 300. KEGG enrichment analysis showed that the 2,274 genes that could correspond to the KEGG pathway are concentrated in 39 metabolic pathways (Figure 2C), and the main pathways are listed in descending order of involved genes by quantity as follows: the ATPase transport metabolic pathway (ko02010; 179 genes), carbohydrate metabolic pathway (ko01200; 134 genes), amino acid biosynthetic metabolic pathway (ko01230; 133 genes).

### Identification of the Active Ingredient in CEL

The previous analysis demonstrated that *S. bikiniensis* HD-087 has five types of NRPS gene clusters for synthesizing antimicrobial lipopeptides. Hence, we attempted to extract lipopeptides from the HD-087 culture filtrates by acid precipitation and alcohol extraction and then analyzed these filtrates by LC–MS using the parameters specific for different CLPs (Płaza et al., 2015; Sun et al., 2019). The LC–MS/MS results confirmed that *S. bikiniensis* HD-087 can produce and secrete at least eight CLPs in three families as follows: three surfactin (1,030 and 1,044 m/z), two fengycin (1,575 and 1,588 m/z) and three iturin (1,045, 1,073 and 1,087 m/z) analogs (Supplementary Figure 1), validating the genome-mining results by antiSMASH prediction. In addition, other lipopeptides in *S. bikiniensis* CEL need to be analyzed in depth in the future.

### Effect of CEL on Spore Germination and Appressorium Formation of *Magnaporthe oryzae*

**Figure 3:**
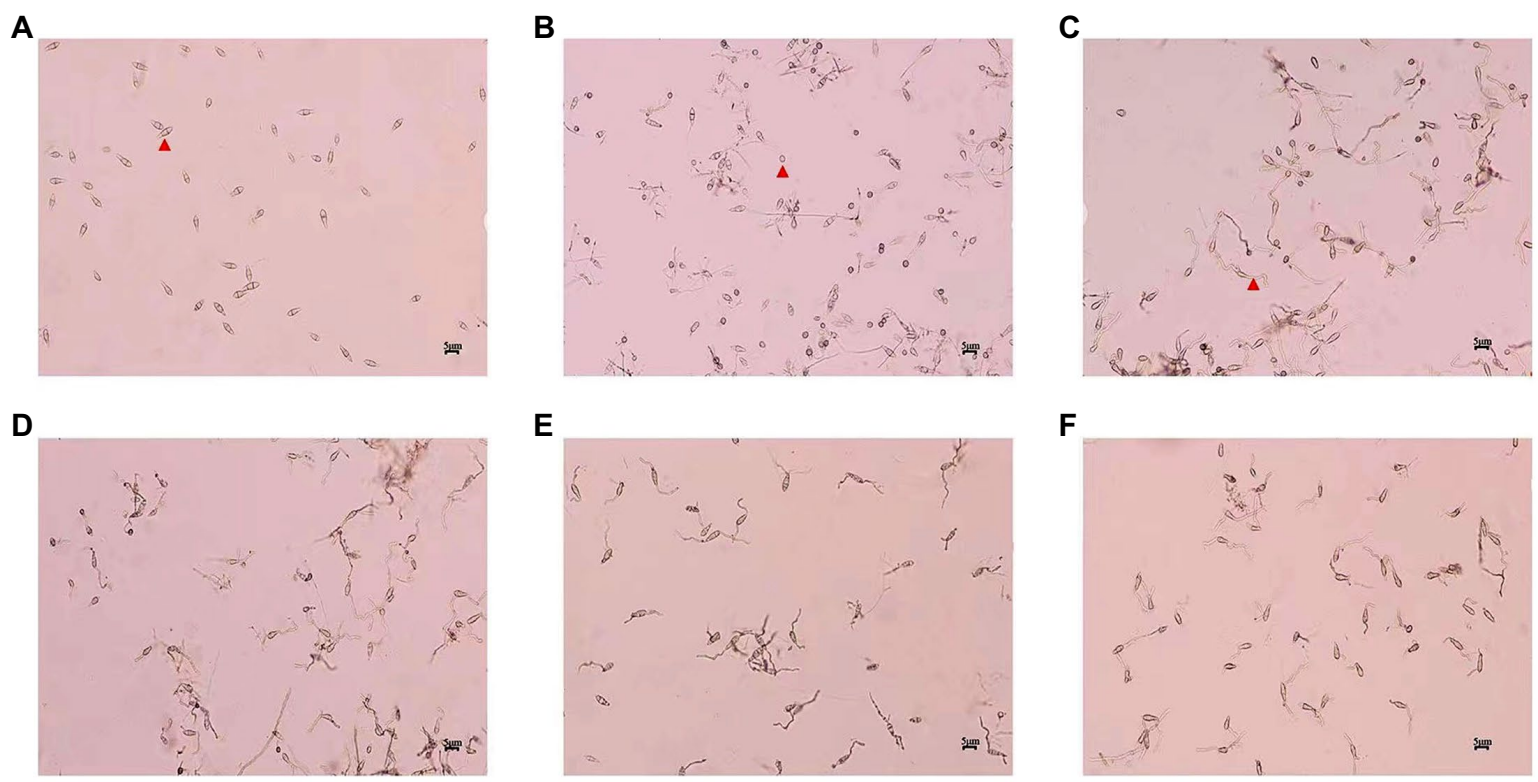
Morphology of spore germination and appressorium formation of *Magnaporthe oryzae* after treatment with crude extract of lipopeptides (CEL). **(A)** Non-CEL-treated (0 h control); **(B–F)** were CEL treated for 6 h at concentrations of 0, 25, 50, 100, and 200 μg·ml^−1^, respectively. Red arrowheads in figures **A–C** show separately the spore, appressorium and germ tube.

*Magnaporthe oryzae* spores from the control group germinated and formed appressoria at 6 h, and a large number of mycelia grew after 24 h. The spore germination and appressorium formation of those treatments with different concentrations of CEL were clearly inhibited (Table 1; Figure 3). The percentage of spore germination and appressorial formation gradually decreased with increased CEL concentration. The spore germination and appressorial formation was calculated by regression equation of virulence; the EC_50_ values of CEL for the inhibition of spore germination and appressorium formation were 29.34 μg·ml^−1^ and 6.95 μg·ml^−1^, respectively. These results indicate that the *S. bikiniensis* HD-087 CEL could be a highly suitable inhibitory agent against *M. oryzae*.

**Table 1:** Effect of CEL on spore germination and appressorium formation of *Magnaporthe oryzae*.

CEL concentration (μg·ml^−1^)	Spore germination rate (%)	Inhibition rate of spore formation (%)	Appressorium formation rate (%)	Inhibition rate of appressorium formation (%)
0	52.23 ± 1.13e	—	43.4 ± 3.32d	—
25	27.5 ± 0.5c	47.2	10.5 ± 0.32c	74.9
50	21.84 ± 2.12d	58.27	6.86 ± 0.5bc	84.2
100	16.04 ± 0.95b	69.3	5.64 ± 0.76ab	93.3
200	11.63 ± 1.42a	76.7	1.5 ± 0.65a	96.6
Regression equation	*y* = 0.8968x + 3.6838		*y* = 1.3129x + 3.8218	
EC_50_ (μg·ml^−1^)	29.34		6.95	

Different small letters indicated significant different among samples (p < 0.05).

### Effect of CEL on *Magnaporthe oryzae* Cell Membrane Integrity

**Figure 4:**
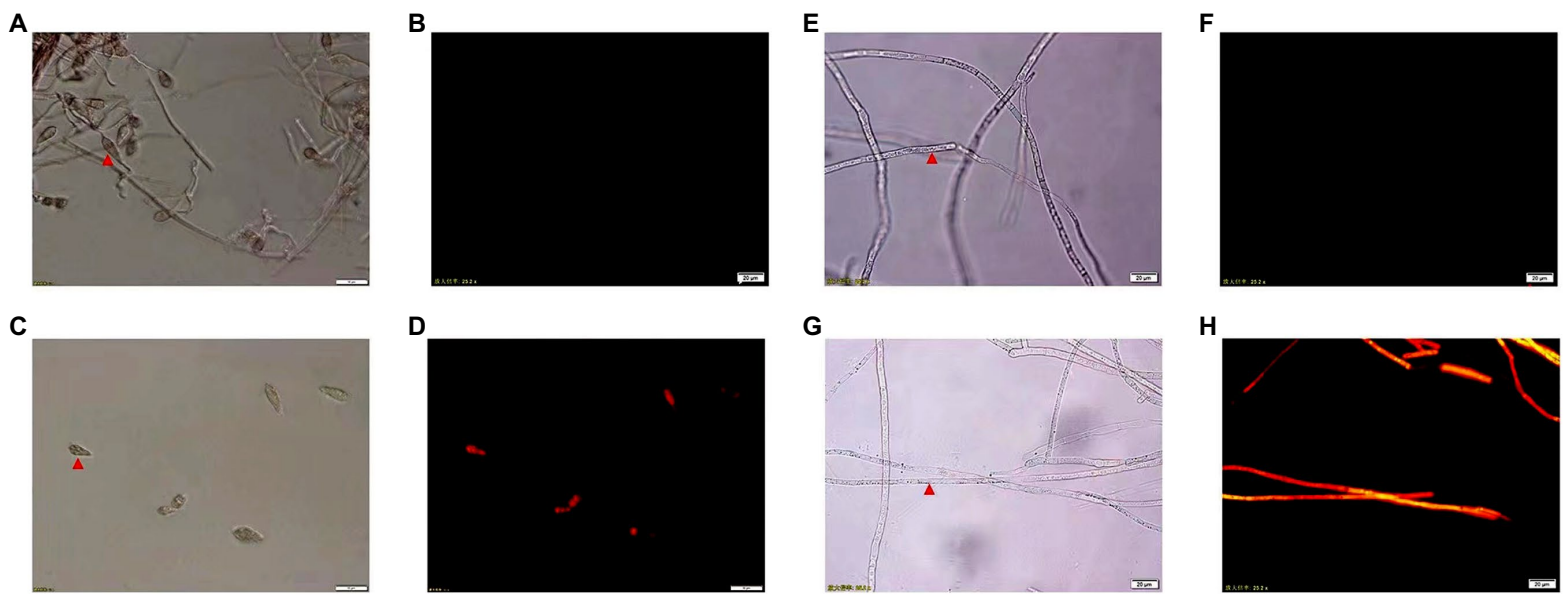
Morphology of spores and mycelium of *Magnaporthe oryzae* under fluorescence microscope. **(A)** Non-CEL-treated spores under white light; **(B)** non-CEL-treated spores under fluorescent light; **(C)** CEL-treated spores under white light; **(D)** CEL-treated spores under fluorescent light; **(E)** non-CEL-treated mycelium under white light; **(F)** non-CEL-treated mycelium under fluorescent light; **(G)** CEL-treated mycelium under white light; and **(H)** CEL-treated mycelium under fluorescent light. The red arrowheads indicate spores and hyphae.

The CEL’s effects on *M. oryzae* cell membrane integrity are shown in Figure 4. PI cannot pass through the living cell membrane but can pass through a damaged cell membrane and stain the nucleus. The fluorescence microscope observations indicated that the spore and mycelium of *M. oryzae* treated with CEL emitted red fluorescence, but that of the control did not. These results demonstrated that the CEL of *S. bikiniensis* can destroy the integrity and permeability of the *M. oryzae* cell membrane.

### Effect of CEL on *Magnaporthe oryzae* Mycelial Morphology

**Figure 5:**
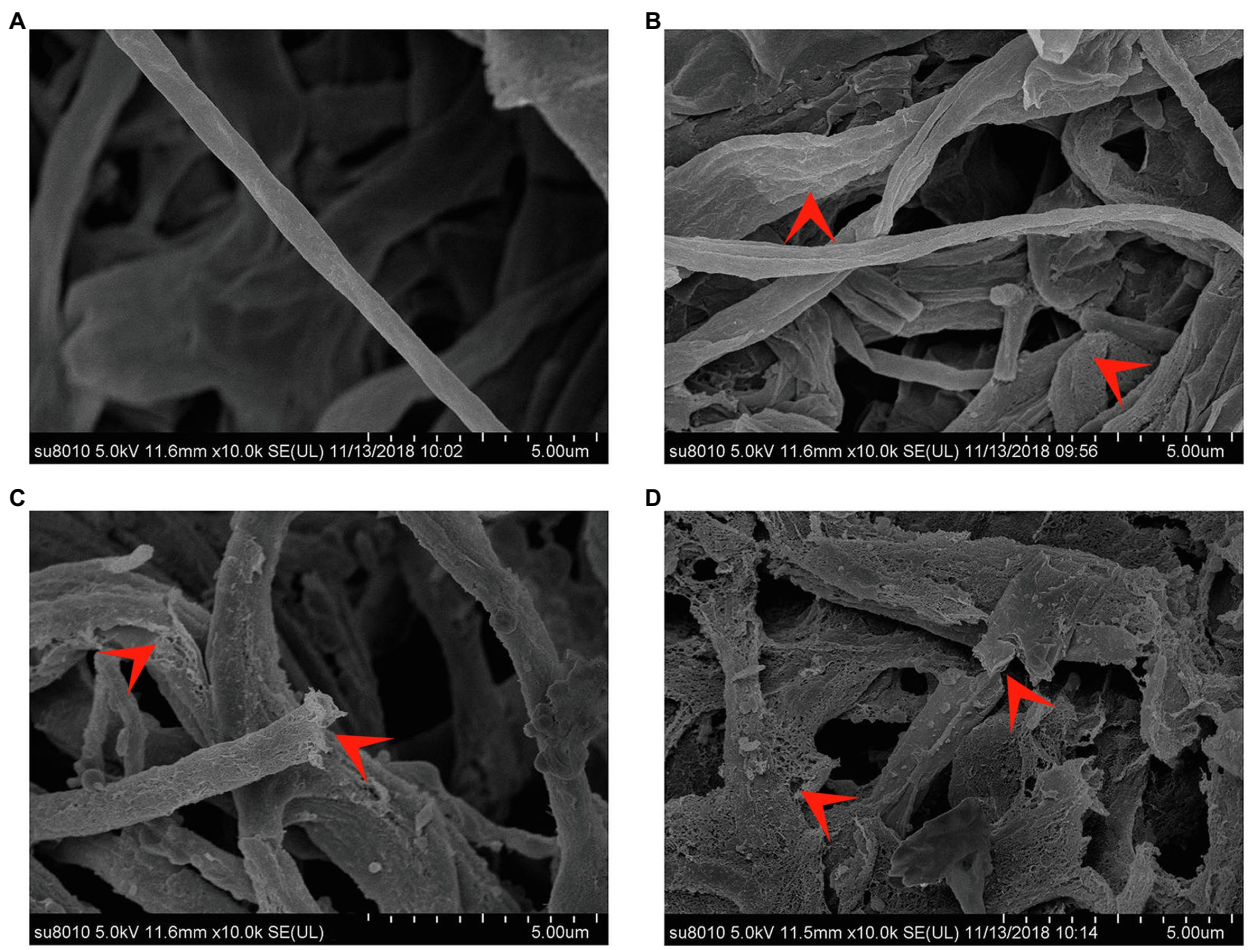
Morphology of *Magnaporthe oryzae* under SEM. **(A)** Non-CEL-treated (control); **(B–D)** CEL treated for 6, 12, and 24 h, respectively. The red arrowheads indicate typical morphology.

Observations under SEM (Figure 5) illustrated that *S. bikiniensis* CEL was able to destroy *M. oryzae* mycelial cell wall integrity. The mycelium of *M. oryzae* without CEL treatment was continuous and stout with round and smooth surfaces. However, on the mycelium treated by CEL for 6 h at the EC_50_ concentration, their surfaces became rough, wrinkled and blistered; after 12 h of treatment, the mycelium was swollen, there was superficial destruction, and some were even fractured; following treatment for 24 h, many reticulated cavities appeared on the mycelium surface and subsequently underwent serious fragmentation. Overall, the cells of *M. oryzae* Guy11 treated with the CEL of *S. bikiniensis* HD-087 exhibited abnormal morphology, suggesting that this CEL degrades the cell wall and destroys the cell membrane.

### Experiments on Controlling Leaf Blast on Rice Leaves *in vitro* by CEL

**Figure 6:**
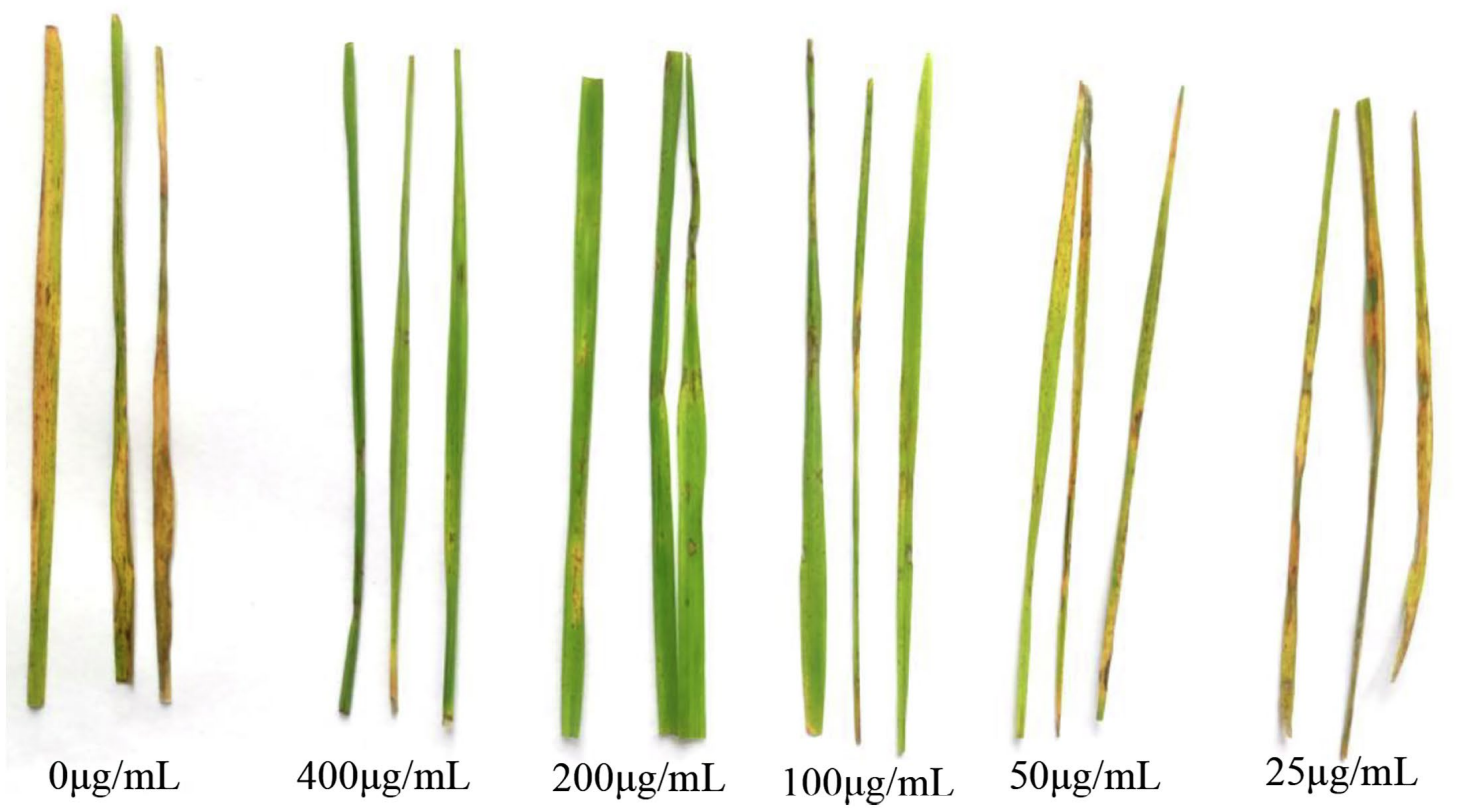
Disease index and control effect of CEL against *Magnaporthe oryzae* at different concentrations.

Table 2 and Figure 6 show that with an increase of CEL concentration of 25–200 μg·ml^−1^, the biocontrol efficacy against rice blast was also improved significantly. However, when the concentration was 400 μg·ml^−1^, the control effect did not rise but decreased, which indicated that an excessive CEL concentration did harm the rice plants. The results showed that 200 μg·ml^−1^ was the optimum concentration of CEL for controlling rice blast, and the best therapeutic effect was up to 70%. On the basis of the above results, 200 μg·ml^−1^ CEL was used to compare the therapeutic effect with commercial biological and chemical pesticides. According to Table 3, the control efficacy of 200 μg·ml^−1^ CEL on leaf blast reached 76.9%; at the same time, the effect of kasugamycin, isoprothiolane, and tricyclazole was far below that of CEL. The results demonstrated that potted rice experiments could be carried out in the next step.

**Table 2:** Disease index and control effect of CEL against *Magnaporthe oryzae* at different concentrations.

CEL concentration (μg·ml^−1^)	Disease index	Biocontrol efficacy (%)
400	50 ± 5.45e	50
200	30 ± 2.32d	70
100	70 ± 1.88c	30
50	83.3 ± 3.12b	16.7
25	100 ± 1.56a	0
Incidence control	100 ± 0.86a	–

Different small letters indicated significant different among samples (p < 0.05).

**Table 3:** Effect of different agents on the treatment of leaf blast of detached rice leaves.

Reagent treatment	Disease index	Biocontrol efficacy (%)
Isoprothiolane	73 ± 2.21c	15.8
Tricyclazole	73 ± 0.46c	15.8
Kasugamycin	55 ± 2.98b	36.6
CEL	20 ± 3.42a	76.9
Incidence control	86.7 ± 3.12d	–

Different small letters indicated significant different among samples (p < 0.05).

### Controlling Leaf Blast on Potted Rice With CEL

**Figure 7:**
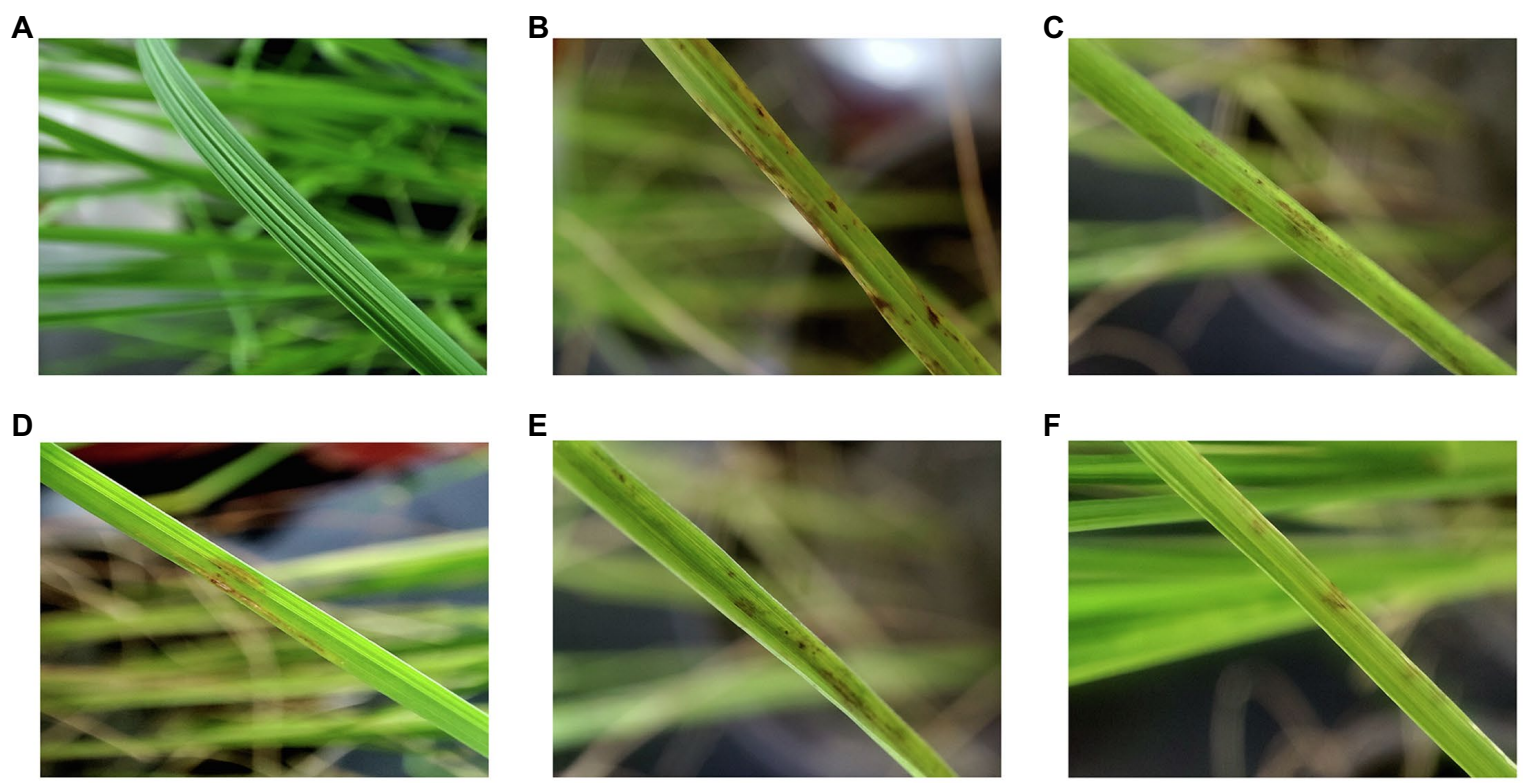
Effect of different agents on the treatment of leaf blast of potted rice. **(A)** Healthy control; **(B)** incidence control; **(C)** tricyclazole-treated; **(D)** kasugamycin-treated; **(E)** isoprothiolane-treated; and **(F)** CEL-treated.

The pot trial results are given in Table 4 and Figure 7. The rice blast disease index of the incidence control group reached 75 ± 2.12, and for the trial groups including CEL, kasugamycin, isoprothiolane, and tricyclazole, this value was significantly reduced. In particular, the disease index of the CEL group fell to 30 ± 0.96; the data were significantly lower than for the abovementioned commercial agents, correspondingly, and the control effect of CEL against rice blast was highest at 60%. At the same time, the control effect of kasugamycin appeared to be relatively superior to tricyclazole and isoprothiolane. These results indicated that the CEL of *S. bikiniensis* HD-087 could inhibit *M. oryzae* infection and reduce the disease index of leaf blast. In addition, compared to the detached leaf experiment, the effect of three commercial agents increased in potted rice, whereas that of the CEL decreased to some degree. This is an interesting observation worthy of further attention.

**Table 4:** Effect of different agents on the treatment of leaf blast of potted rice.

Reagent treatment	Disease index	Control efficacy (%)
Isoprothiolane	52.3 ± 0.62d	30.2
Tricyclazole	41.6 ± 1.08b	44.5
Kasugamycin	35.3 ± 0.86b	52.7
CEL	30 ± 0.96c	60.0
Incidence control	75 ± 2.12a	–

Different small letters indicated significant different among samples (p < 0.05).

## Discussion

*Streptomyces bikiniensis* HD-087 is a soil-derived biocontrol strain against plant pathogenic fungi, including *F. oxysporum* and *M. oryzae*. The whole-genome analysis revealed it to have 19 gene clusters coding secondary metabolites, such as NRPS and PKS gene clusters. Passari et al. (2015) and Sharma et al. (2016) showed that actinomycetes possessing antifungal activity are positively related to NRPS and PKS biosynthetic pathways. The CEL was extracted from the cell-free fermentation liquid of *S. bikiniensis* HD-087 according to gene prediction and was identified by LC–MS/MS. The results demonstrated that this CEL contained surfactins, iturins, and fengycins. Genome-wide comparison revealed that genes involved in fatty acid biosynthesis were highly conserved in all strains, including four encoding 3-oxoacyl-[Acyl-carrier-protein] reductase and seven encoding long-chain fatty acid CoA ligase. It is noteworthy that strain HD-087 boasts five different *FabGs*. *FabG* is involved in fatty acid elongation (Hu et al., 2019). Bacteria harboring different *FabG* alleles produce variable fatty acid chains linked to the cyclic lipopeptides and result in chemical analogs with different bioactivities. The diversity of *fabG* and the CLP synthetase modules enable *Streptomyces* to synthesize multiple CLP isomers with differential functions in a limited genetic capacity, making them able to survive in diverse environments. HD-087 also has the *sfp* gene encoding 4′-phosphopantene transferase, which is crucial for the biosynthesis of CLP and PKS by activating the peptidyl carrier protein (PCP) domain in the CLP modules and the acyl carrier protein (ACP) domain in the PKS modules (Fazle Rabbee and Baek, 2020; Morales-Aparicio et al., 2020).

The genome-wide gene cluster results of *S. bikiniensis* HD-087 also showed that there is a gene cluster for which the sequence is 100% similar to the PTM synthesis gene cluster of *Streptomyces griseus*, which can encode multiple structural antibiotics, including somatostatin, a growth inhibitor against the plant pathogen *F. oxysporum* MHKW and *Alternaria brassicae* BCHB (Hou et al., 2020). There are also iron carrier gene clusters with 100% similarity to the synthetic gene cluster of desferrioxamine B in *S. griseus*, suggesting that *S. bikiniensis* HD-087 has the potential to synthesize the secretory type of iron carriers, and secreted siderophores could suppress the pathogen *in vitro* and protect plants from pathogen infection by changing rhizosphere microbiome members (Gu et al., 2020). Notably, other unmatched gene clusters were also found in the *S. bikiniensis* HD-087 genome, which might participate in the biosynthesis of some key secondary metabolites.

The results of the spore germination test indicated that the key role of CEL in preventing *M. oryzae* infection of rice plants is the inhibition of spore germination and appressorium formation. The results of PI staining and SEM observations further clarified the antifungal mechanism of CEL for destroying the integrity of the mycelium cell wall and the semipermeability of *M. oryzae* spore and mycelial cell membranes. Compared to *Bacillus vallismortis* R2 and *Bacillus megaterium* L2, which secrete fengycins against *A. alternata* by eliciting swollen hyphae (Li et al., 2015; Kaur et al., 2017), CEL showed a better effect regarding fungicidal activity. Wu et al. also recently demonstrated that fengycin B produced by *Bacillus subtilis* PMB102 had antifungal activity against *A. brassicae* ABA-31 by activating swollen mycelia (Wu et al., 2021). It is generally accepted that the action mode of lipopeptides relies on cell wall and membrane disruption, causing leakage of the cytoplasm and cell death (Kaspar et al., 2019).

It also appears that lipopeptides’ action on plant pathogenic fungi are rather more complex. The most recent studies have shown that lipopeptides significantly reduce the expression of some genes involved in autophagy in *M. oryzae* (Zhang et al., 2022). Yin et al. reported that the autophagy pathway directly affects spore germination and appressorium formation, thus influencing *M. oryzae* pathogenicity (Yin et al., 2019). The CWI (cell wall integrity) maintenance mechanism was found to be a key factor in the pathogenicity of *M. oryzae* (Feng et al., 2021), and there is crosstalk between CWI and autophagy pathways (Yin et al., 2019). However, how is lipopeptide damage to the cell wall of *M. oryzae* related to autophagy? How does this damage affect autophagy and CWI maintenance? These questions remain to be explored in depth.

To explore the biocontrol potential of *S. bikiniensis* HD-087 lipopeptides further, we used experiments on the control of leaf blast on rice leaves *in vitro* and potted rice by CEL to lay a strong foundation for further field tests on controlling rice blast. A number of other researchers provided similar results; for example, Ma et al. (2020) showed that both 10 and 50 μg·ml^−1^ lipopeptides produced by *Bacillus velezensis* 11-5 were effective against the occurrence of leaf blast on detached rice leaves (Ma et al., 2020). However, as found in this experiment, the control effect of CEL against rice leaf blast on potted rice seedlings was much less than that on detached leaves, and we have not yet been able to demonstrate whether CEL can maintain the control efficacy at 60% in plot or field trials. Of course, we will soon investigate this question.

Based on the genome-wide analysis of *S. bikiniensis* HD-087, two research tasks can be performed in the future: First, to exploit the potential of the antimicrobial gene cluster and obtain new antimicrobial substances through bioinformatics and combinatorial biology studies; second, to deeply analyze the known metabolic synthesis pathways and their influencing factors to ultimately improve their yield through genetic manipulation.

## Conclusion

In this study, lipopeptides predicted by antiSMASH analysis of the functional genome were successfully extracted from a fermentation broth of *S. bikiniensis* HD-087. Surfactins, iturins and fengycins were all found to exist in the crude extract of lipopeptides by LC–MS/MS. The control effects of *S. bikiniensis* HD-087 lipopeptides against *M. oryzae* were noticeable according to *in vivo* and *in vitro* tests and could certainly be applied as biocontrol agents to control rice blast in plot and field experiments. As safe, natural substances, these compounds would be appropriate competitive substitutes for the various highly toxic chemical pesticides in controlling rice blast. Overall, this study highlights the feasibility of using genomic analysis to predict and develop biocontrol pharmaceuticals, and this method could accelerate the research process for new drugs.

## Data Availability Statement

The data presented in the study are deposited in the National Center for Biotechnology Information NCBI Gene Bank repository, accession number PRJNA823498. The datasets presented in this study can be found in online repositories. The names of the repository/repositories and accession number(s) can be found at: https://www.ncbi.nlm.nih.gov/genbank.

## Author Contributions

WL: conceptualization, methodology, data curation, writing original draft, formal analysis, and writing—review and editing. JW: conceptualization, methodology, data curation, formal analysis, and visualization. SL: data curation. HZ and LL: methodology. LM: visualization. WP: conceptualization and project administration. CD: resources, supervision, and funding acquisition. All authors contributed to the article and approved the submitted version.

## Funding

This research was supported by the National Natural Science Foundation of China (project no. 32172468).

## Conflict of Interest

The authors declare that the research was conducted in the absence of any commercial or financial relationships that could be construed as a potential conflict of interest.

## Publisher’s Note

All claims expressed in this article are solely those of the authors and do not necessarily represent those of their affiliated organizations, or those of the publisher, the editors and the reviewers. Any product that may be evaluated in this article, or claim that may be made by its manufacturer, is not guaranteed or endorsed by the publisher.
