# Supplementary material for: Genomic and Biocontrol Potential of the Crude Lipopeptide by Streptomyces bikiniensis HD-087 Against Magnaporthe oryzae
Source: Front Microbiol. 2022 Jun 9;13:888645. doi: 10.3389/fmicb.2022.888645 (PMC9218715; doi:10.3389/fmicb.2022.888645)
Supplement: Supplementary file 1 [file Table_1.DOCX]

| Gene cluster type | Starting position | Termination position | Gene numbers | The most similar known gene cluster | Proportion of similar genes |
| --- | --- | --- | --- | --- | --- |
| terpene-nrps | 170375 | 257007 | 77 | Lividomycin biosynthetic gene cluster from *S. lividus* | 10% |
| t1pks-nrps | 385849 | 435257 | 34 | SGR PTMs biosynthetic gene cluster from *S. griseus* | 100% |
| terpene | 468965 | 495520 | 28 | Hopene biosynthetic gene cluster from *S. coelicolor* | 76% |
| bacteriocin | 573465 | 583680 | 7 | - | - |
| bacteriocin | 989405 | 1000793 | 12 | - | - |
| lantipeptide | 1101289 | 1125878 | 25 | Kanamycin biosynthetic gene cluster from *S. kanamyceticus* | 1% |
| siderophore | 1412787 | 1427859 | 13 | - | - |
| terpene | 1677102 | 1699387 | 26 | Geosmin biosynthetic gene cluster from *S. coelicolor* | 100% |
| terpene | 2020431 | 2041481 | 20 | Albaflavenone biosynthetic gene cluster from *S. coelicolor* | 100% |
| thiopeptide | 2539352 | 2571836 | 27 | - | - |
| lantipeptide | 2852884 | 2893051 | 38 | SAL-2242 biosynthetic gene cluster from *S. albus* | 100% |
| lassopeptide | 3124704 | 3146989 | 19 | Kanamycin biosynthetic gene cluster from *S. kanamyceticus* | 7% |
| nrps | 3207586 | 3269818 | 40 | Desferrioxamine B biosynthetic gene cluster from *S. griseus* | 11% |
| bacteriocin | 3840918 | 3851362 | 12 | Desferrioxamine B biosynthetic gene cluster from *S. griseus* | 6% |
| nrps | 3904566 | 4010261 | 45 | Mannopeptimycin biosynthetic gene cluster from *S. hygroscopicus* | 7% |
| siderophore | 4720355 | 4732173 | 11 | Desferrioxamine B biosynthetic gene cluster from *S. griseus* | 100% |
| ectoine | 5633681 | 5644077 | 11 | Ectoine biosynthetic gene cluster from *S. anulatus* | 100% |
| bacterioc in-terpene | 6431667 | 6463892 | 39 | Isorenieratene biosynthetic gene cluster from *S. griseus* | 85% |
| t3pks-lantipepti de-t1pks-nrps | 6559707 | 6871738 | 155 | FR-008 biosynthetic gene cluster from *S. sp.* FR-008. | 100% |

Table S.1 Gene cluster of secondary metabolite synthesis of *S. bikiniensis* HD-087
