# Supplementary figures and images for: Genomic and Biocontrol Potential of the Crude Lipopeptide by Streptomyces bikiniensis HD-087 Against Magnaporthe oryzae
Source: Front Microbiol. 2022 Jun 9;13:888645. doi: 10.3389/fmicb.2022.888645 (PMC9218715; doi:10.3389/fmicb.2022.888645)

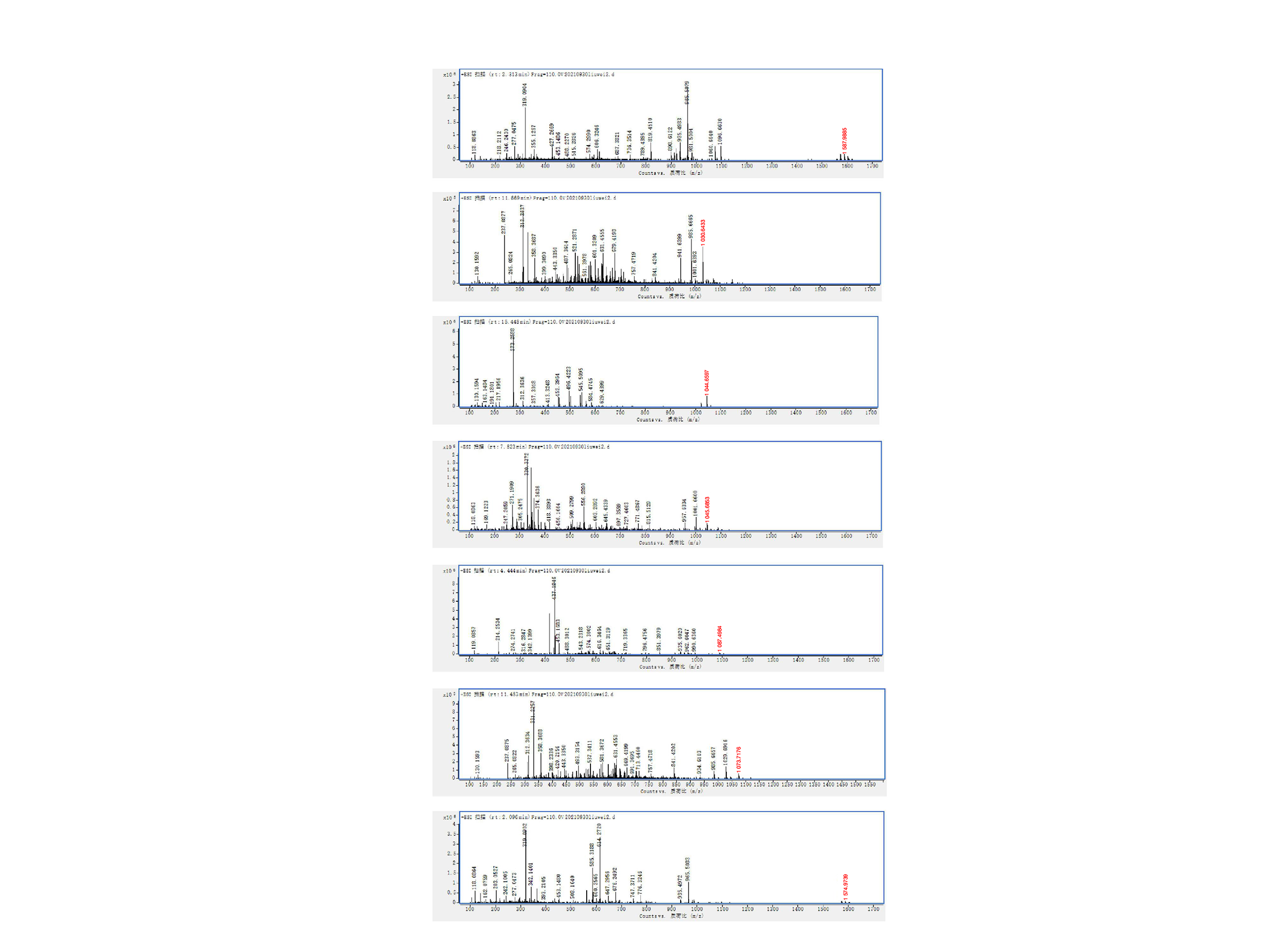

Supplement: Supplementary file 2 [file Image_1.TIF]
